# SH2 Domain-Containing Phosphatase-SHP2 Attenuates Fibrotic Responses through Negative Regulation of Mitochondrial Metabolism in Lung Fibroblasts

**DOI:** 10.3390/diagnostics13061166

**Published:** 2023-03-18

**Authors:** Theodoros Karampitsakos, Apostolos Galaris, Ilianna Barbayianni, Giuseppe DeIuliis, Farida Ahangari, Fotis Sampsonas, Vasilina Sotiropoulou, Vassilis Aidinis, Anton M. Bennett, Jose D. Herazo-Maya, Nikolaos Xylourgidis, Petros Bakakos, Demosthenes Bouros, Naftali Kaminski, Argyrios Tzouvelekis

**Affiliations:** 1 Department of Respiratory Medicine, University Hospital of Patras, 26504 Patras, Greece; 2 Biomedical Sciences Research Center “Alexander Fleming”, 16672 Athens, Greece; 3 Department of Internal Medicine, Pulmonary, Critical Care and Sleep Medicine, Yale School of Medicine, New Haven, CT 06520, USA; 4 Department of Pharmacology, Yale School of Medicine, New Haven, CT 06520, USA; 5 Ubben Center and Laboratory for Pulmonary Fibrosis Research, Morsani College of Medicine, University of South Florida, Tampa, FL 33620, USA; 6 Medical School, National and Kapodistrian University of Athens, 15784 Athens, Greece

**Keywords:** SHP2, mitochondrial metabolism, mammalian target of rapamycin complex-1, fibroblast

## Abstract

Background: We have previously shown that SHP2 downregulation may predispose fibroblasts to differentiate into myofibroblasts and proposed a role for SHP2 downregulation in the pathogenesis of idiopathic pulmonary fibrosis (IPF). Recent data have shown that SHP2 localizes to the mitochondrial intercristae, and its overexpression enhances mitochondrial metabolism leading to oxidative stress and senescence. Objective: To determine the effect of SHP2 on fibrotic responses. Methods and Results: Primary mouse lung fibroblasts derived from mice carrying a conditional knock-in mutation (D61G/+), rendering the SHP2 catalytic domain constitutively active, had reduced proliferation (1.6-fold, *p* < 0.05), migration (2-fold, *p* < 0.05), as well as reduced responsiveness of TGFB-1 induced fibroblasts-to-myofibroblasts differentiation, compared to wild-type ones. Electron microscope analysis revealed that SHP2 ^D61G/+^ mouse lung fibroblasts were characterized by mitochondrial abnormalities, including swollen mitochondria with disrupted electron-lucent cristae and an increased number of autophagosomes compared to wild-type ones. SHP2 ^D61G/+^ MLFs exhibited increased protein levels of autophagy markers, including LC3B-II and p-62, evidence that was confirmed by immunofluorescence analysis. Mitochondrial function analysis revealed that stable (genotype D61G/+) overexpression of SHP2 led to impaired mitochondrial function, as assessed by decreased mitochondrial membrane potential (1.29-fold, *p* < 0.05), coupling efficiency (1.82 fold, *p* < 0.05), oxygen consumption rate (1.9-fold, *p* < 0.05), and increased reactive oxygen species production both at baseline (1.75-fold, p < 0.05) and following H_2_O_2_ stimulation (1.63-fold, *p* < 0.05) compared to wild-type ones (SHP2^+/+^). SHP2 ^D61G/+^ mouse lung fibroblasts showed enhanced AMPK activity, as well as decreased activation of the mTORC1 signaling pathway, potentially leading to ineffective mitochondrial metabolism and increased autophagy. Conclusions: SHP2 attenuates fibrotic responses in fibroblast cell lines through negative regulation of mitochondrial metabolism and induction of autophagy. SHP2 activation may represent a promising therapeutic strategy for patients with fibrotic lung diseases.

## 1. Introduction

Interstitial lung diseases (ILDs) encompass a heterogenous group of chronic and potentially progressive lung diseases. ILDs are characterized by lung architectural distortion, with a variable amount of fibrotic and/or inflammatory lesions as a response to injurious stimuli to alveolar epithelial cells [1,2]. Despite extensive research efforts and several randomized controlled trials, the identification of novel compounds that could halt disease progression have lagged behind, and thus mortality is still high [2,3,4]. Currently, nintedanib is a first-line treatment option for progressive pulmonary fibrosis, while further research has been suggested for pirfenidone1. Both compounds have been approved for IPF and seem to slow down lung function decline, yet leave patients with major functional disability and poor quality of life [5,6,7,8,9,10].

Given the fruitful effects derived from the investigation of tyrosine kinases and tyrosine kinase inhibitors in pulmonary fibrosis, studying tyrosine phosphatases seems rational. Moreover, focusing on deregulated metabolism and autophagy might be crucial for the development of novel anti-fibrotic compounds. Reduced autophagy is associated with aging. Both deregulated autophagy and aging seem to have a cardinal role in the pathogenesis of pulmonary fibrosis [11].

Our study group has previously shown that SHP2, a ubiquitously expressed non-receptor tyrosine phosphatase, was downregulated in IPF lungs, and when overexpressed, it attenuated fibrosis through negative regulation of pro-fibrotic kinase-related signal transduction pathways leading to impairment of fibroblast homeostasis [12]. Furthermore, we and others have identified the key role of mitochondrial dysfunction in the context of alveolar epithelial cell injury and apoptosis in pulmonary fibrosis that could be reduced by exogenous administration of the thyroid hormone and its analogues, leading to the resolution of lung fibrosis and inflammation in murine models, thus making it a potential therapeutic target [13,14,15]. SHP2 has been reported to lie within the intercristae/intermembrane space of mitochondria [16,17,18]. In particular it has been demonstrated that SHP2 regulates a complex unit of enzymes involved in oxidative phosphorylation in mouse embryonic fibroblasts [19]. To this end, we hypothesized that our previously published data showing SHP2 as an anti-fibrotic mediator in the context of lung fibrosis [12] could be partially explained by the negative regulation of fibroblast mitochondrial homeostasis, leading to ineffective mitochondrial metabolism and disrupted fibrotic responses.

To this end, we used primary mouse lung fibroblasts (MLFs) derived from mice carrying a conditional knock-in mutation (D61G/+) that renders the SHP2 catalytic domain constitutively active, and we validated the previously published observation that SHP2 activation is associated with attenuated fibrotic responses in vitro, as assessed by reduced migration, differentiation, and proliferation in response to pro-fibrotic stimuli. When we used transmission electron microscopy to examine mitochondria from SHP2^D61G/+^ constitutively active mutants, we noticed several morphological abnormalities, including swollen mitochondria with disrupted, electro-lucent cristae and an increased number of autophagosomes, resulting in impaired mitochondrial metabolism and increased production of ROS. Intriguingly, we demonstrated for the first time that SHP2 impaired mitochondrial metabolism of MLFs through mechanisms that involved negative regulation of mTORC1-signaling machinery and induction of Ulk1-AMPK interaction, thus activating the autophagy machinery [20].

## 2. Materials and Methods

### 2.1. Protein Extraction and Immunoblot Analysis

Total protein extracts were isolated from the cells upon mentioned treatments using common procedures, transferred into PVD-membranes, and hybridized overnight using the following primary antibodies (PINK1-ab23707, LC3B-ab51520, β-actin-sc-47778, and p62/SQSTM1-ab56416) obtained from Santa Cruz Biotechnology, CA (sc), or Abcam, Cambridge, UK (ab).

### 2.2. Proliferation Assay

The Quick Cell Proliferation Assay Kit (Abcam-ab65473) was used to measure primary MLFs from SHP2^D61G/+^ and SHP2^+/+^ mice proliferation, as it was previously described [21].

### 2.3. Immunofluorescence Staining

Primary MLFs from SHP2^D61G/+^ and SHP2^+/+^ mice were plated at a density of 0.5 × 10^6^ cells/well on coverslips on 8-well culture dishes. After TGFB1 (10 ng/mL) stimulation of the cells for 6 h, we followed the previously described protocol [12,21].

### 2.4. Wound Healing (Scratch Assay)

The wound healing (or scratch) assay was used for the measurement of two-dimensional cell migration. An artificial gap was created on a confluent cell monolayer and subsequently, movement tracked through microscopy/other imaging. Primary MLFs from SHP2^D61G/+^ and SHP2^+/+^ mice were seeded in 24-well plates at 2 × 10^5^ cells/mL and incubated for 24 h. The previously published established protocol was followed [22].

### 2.5. Extracellular Flux Technology

The oxygen consumption rates (OCR-maximum respiration) of primary MLFs from SHP2^D61G/+^ and SHP2^+/+^ mice were measured by using a Seahorse XF96 Extracellular Flux Analyzer (Seahorse Bioscience, Billerica, MA, USA), as previously described [13,23].

### 2.6. Determination of ATP Production

Total ATP was measured using an ATP Fluorometric Assay Kit (BioVision, San Francisco, CA) as previously described [13,23].

### 2.7. Mitochondrial Membrane Potential (MMP)

The alterations in relative MMP (ΔΨm) were determined through a JC-10 (5,5′,6,6′-tetrachloro-1,1′,3,3′-tetraethyl-benzamidazolocarbocyanine iodide) molecular probe, based on the protocol previously described [13,23].

### 2.8. Transmission Electron Microscopy (TEM)

With regards to TEM, primary MLFs from SHP2^D61G/+^ and SHP2^+/+^ mice were plated at a density of 0.5 × 10^6^ cells/well on coverslips on 2-well culture dishes and perfused with 4% paraformaldehyde (PFA) and pulmonary samples were dissected out in 2% PFA. Subsequently, we followed the previously described protocol [13]. Images were obtained through Morada CCD and iTEM (Olympus) software.

### 2.9. Measurements of Reactive Oxygen Species (ROS)

The ROS-sensitive probe 2′,7′-dichlorodihydrofluorescein diacetate (H2DCFDA; D-399, Molecular Probes) was used (final concentration:8 μM) for 25 min at 37 °C in the dark. Centrifugation, supplementation of cells with media, and then incubation in the dark for 25 min at 37 °C was performed. As a control, cells were incubated in 100 uM H_2_O_2_. Cells were collected following centrifugation, resuspended in PBS, then transferred into a 96-well plate, and analyzed through an Ascent Fluoroskan plate reader (488 nm excitation; 527 nm emission) as previously presented with slight modifications [19].

### 2.10. Statistical Analysis

Data were statistically analyzed using Med. Calc. version 14. We used the Mann–Whitney U test or unpaired t-test for comparisons between two groups and one-way ANOVA for three or more groups. Data are presented as mean ± SD. Results were considered significant if *p* < 0.05, unless otherwise indicated.

## 3. Results

**Figure 1:**
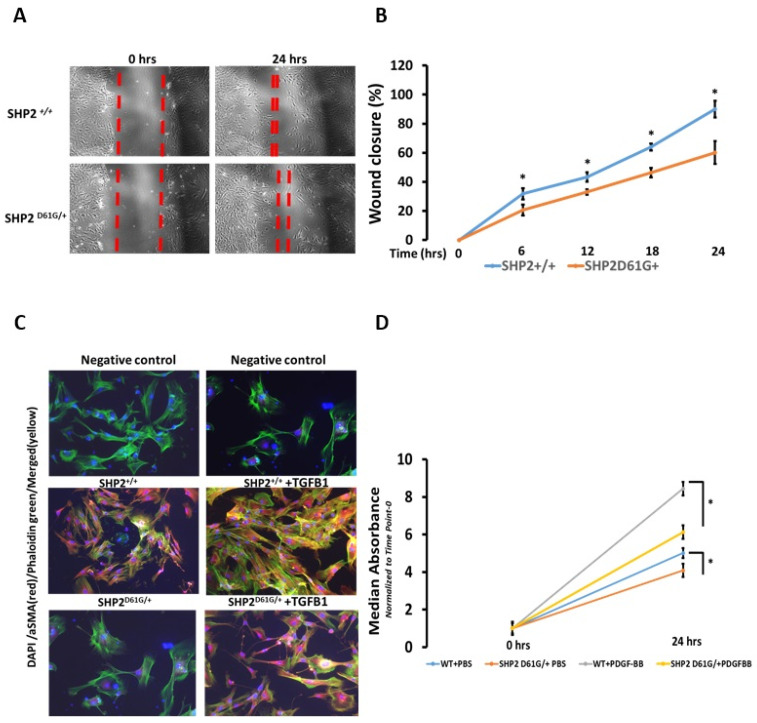
SHP2^D61G/+^ constitutively active mutant negatively regulates fibroblast migration, myofibroblast differentiation, and proliferation. (**A**,**B**) SHP2^D61G/+^ constitutively active mutant lung fibroblasts exhibited significantly reduced cell migration at all time points (T0-T24, 2-fold) compared to wild-type ones (SHP2^+/+^). One way ANOVA, * *p* < 0.05. (**C**) Double immunofluorescence analysis in representative primary MLF samples showing decrease in TGFB1-induced a-SMA (red) and stress fibers as indicated by phalloidin green (merged-yellow) in SHP2^D61G/+^ constitutively active mutant lung fibroblasts compared to wild-type ones (SHP2^+/+^). (**D**) PDGF-BB stimulation induced a significant increase (1.4-fold) in proliferation rates of SHP2^+/+^ MLFs compared to the SHP2^D61G/+^. Each bar represents mean expression of 6 samples (biological replicates). Data (absorbance) represent mean + SD. One-way ANOVA, * *p* < 0.05. Details for this figure are presented in Supplemental Data S1.

The SHP2 ^D61G/+^ constitutively active mutant negatively regulates fibroblast migration, myofibroblast differentiation, and proliferation. We have previously shown that primary MLFs isolated from SHP2^D61G/+^ or normal human lung fibroblasts (NHLFs) with a constitutively active SHP2 mutant (E76A) display attenuated in-vitro fibrotic responses to TGFB1 stimulation [12]. We isolated primary MLFs both from SHP2^D61G/+^ and SHP2^+/+^ mice and investigated their response to pro-fibrotic stimuli (TGFB1, PDGF-BB) in vitro, as well as their spontaneous migratory capacity (scratch assay). SHP2^D61G/+^ lung fibroblasts exhibited significantly reduced cell migration at all time points (T_0_-T_24_, 2-fold, *p* < 0.05) of scratch assay (wound closure) compared to wild-type ones (SHP2^+/+^) (Figure 1A,B, Supplemental Data S1). Furthermore, double immunofluorescence analysis in representative primary MLF samples showed that SHP2 constitutive activation mediated significant decreases in TGFB1-induced a-SMA (red) and stress fibers as indicated by phalloidin green (merged-yellow) compared to wild-type ones (SHP2^+/+^) (Figure 1C). Finally, SHP2^D61G/+^ MLFs exhibited reduced proliferation rates both spontaneously (1.22-fold) and following PDGF-BB (25 ng/mL for 6 h) (1.4-fold) compared to wild-type ones (SHP2^+/+^ ) (Figure 1D). The above findings demonstrate that SHP2 negatively regulates fibroblast homeostasis in vitro.

**Figure 2:**
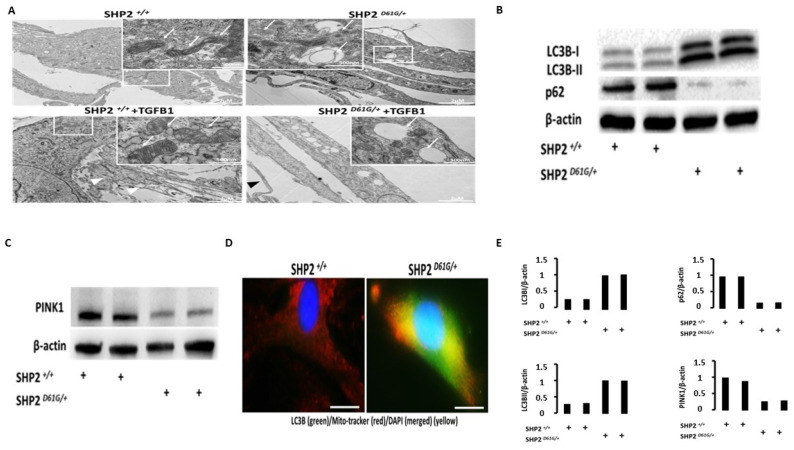
SHP2^D61G/+^ constitutively active mutant MLFs display increased expression of autophagy markers, swollen mitochondria with disrupted cristae, and an increased number of autophagosomes. (**A**) Transmission electron microscopy (TEM) of SHP2^D61G/+^ showed that SHP2 constitutive activation was associated with an increased number of autophagosomes and swollen mitochondria with disrupted, electro-lucent cristae (white arrows-insets). (**B**) Immunoblot analyses of autophagy markers (LC3BI/II) and autophagy receptors (p62) showed a LC3B^high^p62^low^ immunoblot pattern in SHP2^D61G/+^ constitutively active mutant MLFs compared to wild-type ones (SHP2^+/+^), indicating increased autophagy. (**C**) Double immunofluorescence analysis in MLFs validated immunoblot results showing co-localization (yellow-merged) of autophagy marker LC3B (green) with MitoTracker (red stain of active mitochondria) in SHP2^D61G/+^ constitutively active mutant MLFs. (**D**) Immunoblot analysis of PINK1 (mediator of effective autophagy) showed decreased expression in SHP2^D61G/+^ constitutively active mutant MLFs compared to wild-type ones (SHP2^+/+^), potentially indicating ineffective autophagy. (**E**) Immunoblot densitometry analysis of LC3BI/II, p62, and PINK1 normalized to β-actin. Data are presented as bar graphs. Each bar represents an individual lane. Details for this figure are presented in Supplemental Data S2.

SHP2^D61G/+^ constitutively active mutant MLFs display abnormal mitochondria with disrupted, electro-lucent cristae, an increased number of autophagosomes, and upregulation of autophagy markers. After further validating our previously published results [12], we aimed to identify additional mechanisms underlying the already known anti-fibrotic properties of SHP2. Considering that: (1) SHP2 is not only cytoplasmic but also localizes to the intercristae/intermembrane space of mitochondria, (2) mitochondrial metabolism and mitophagy of alveolar type II epithelial cells and fibroblasts play a cardinal role in lung fibrosis [13,24], and (3) SHP2 is a negative regulator of fibroblast homeostasis through dephosphorylation/deactivation of tyrosine-kinase and serine/threonine-kinase fibrotic signal transduction pathways [12]; yet it is currently unknown whether SHP2 may exert its anti-fibrotic properties through negative regulation of fibroblasts in mitochondrial metabolism. To this end, we isolated primary MLFs from SHP2^D61G/+^ constitutively active mutant mice and wild-type ones and performed transmission electron microscopy (TEM) analysis before and after treatment with TGFB1 (10 ng/mL for 6 h). We demonstrated that MLFs from SHP2 knock-in mice exhibited several mitochondrial abnormalities including swollen mitochondria with disrupted, electro-lucent cristae (white arrows-insets) and increased number of autophagosomes. Intriguingly, TEM analyses revealed that SHP2^D61G/+^ MLFs displayed relatively shrunken cytoplasm and an absence of extracellular matrix production compared to wild-type MLFs (SHP2^+/+^), where the presence of secreted extracellular material, following treatment with TGFB1, was evident (white arrow heads) in wild-type MLFs. To enrich our observations, we performed immunoblot and immunofluorescence analysis of autophagy markers (LC3B1/II, p62) and revealed a LC3B^high^p62^low^ immunoblot pattern in SHP2^D61G/+^ constitutively active mutant MLFs compared to wild-type ones (SHP2^+/+^), indicating increased autophagy. In addition, double immunofluorescence analysis in MLFs validated immunoblot results showing co-localization (yellow-merged) of autophagy marker LC3B (green) with MitoTracker (red stain of active mitochondria) in SHP2^D61G/+^ constitutively active mutant MLFs. Surprisingly immunoblot analysis of PINK1 (master regulator of effective autophagy) showed decreased expression in SHP2^D61G/+^ constitutively active mutant MLFs compared to wild-type ones (SHP2^+/+^), potentially indicating ineffective autophagy. The latter observations indicate an association between mitochondrial abnormalities and attenuated fibrotic responses driven by SHP2 constitutive activation in MLFs (Figure 2, Supplemental Data S2).

**Figure 3:**
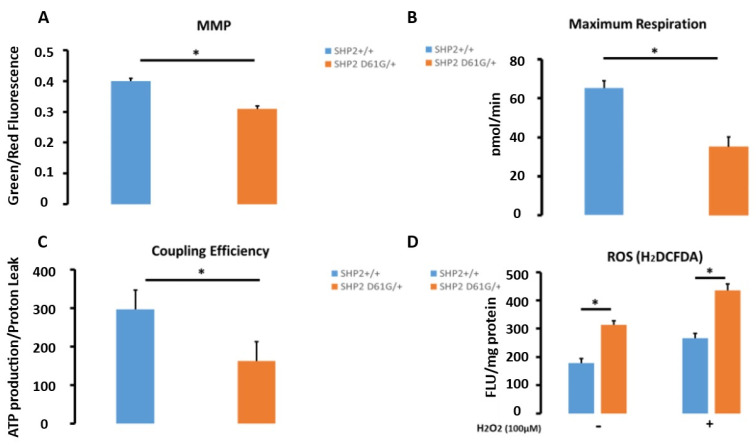
SHP2 D61G/+ constitutively active mutant MLFs exhibit impaired mitochondrial function with increased ROS production. Figure presents (**A**) Mitochondrial function of SHP2 ^D61G/+^, (**B**) Maximum respiration, (pmol/min), (**C**) Coupling efficiency (ATP production/proton leak), and **D**) Reactive oxygen species (ROS). Compared to wild-type ones, D61G cells show a 1.6-fold increased fluorescence, indicating significantly increased ROS levels. Note that SHP2^D61G/+^ constitutively active MLFs were characterized by reduced MMP levels (1.3-fold), maximum respiration (oxygen consumption rate) (1.86-fold), and coupling efficiency (1.9-fold), indicating ineffective mitochondrial metabolism potentially resulting in increased ROS (1.6-fold) compared to wild-type ones. Data are presented as bar graphs with horizontal bars representing mean mitochondrial membrane potential (MMP) levels (green/red fluorescence ratio) ±SEM of 6 samples (biological replicates). Independent samples Student’s *t*-test, * *p <* 0.05. Details for this figure are presented in Supplemental Data S3.

SHP2^D61G/+^ MLFs exhibit impaired mitochondrial function with increased ROS production. To further enrich our previous findings that SHP2 activation promotes mitochondrial abnormalities in primary MLFs, we next investigated whether these are associated with functional abnormalities in SHP2^D61G/+^ constitutively active MLFs and wild-type ones. SHP2^D61G/+^ constitutively active MLFs were characterized by reduced mitochondrial membrane potential (MMP) levels (1.3-fold), maximum respiration (oxygen consumption rate) (1.86-fold), and coupling efficiency (1.9-fold), indicating ineffective mitochondrial metabolism. Intriguingly, D61G cells show a 1.6-fold increase in ROS levels (Figure 3A–D, Supplemental Data S3).

**Figure 4:**
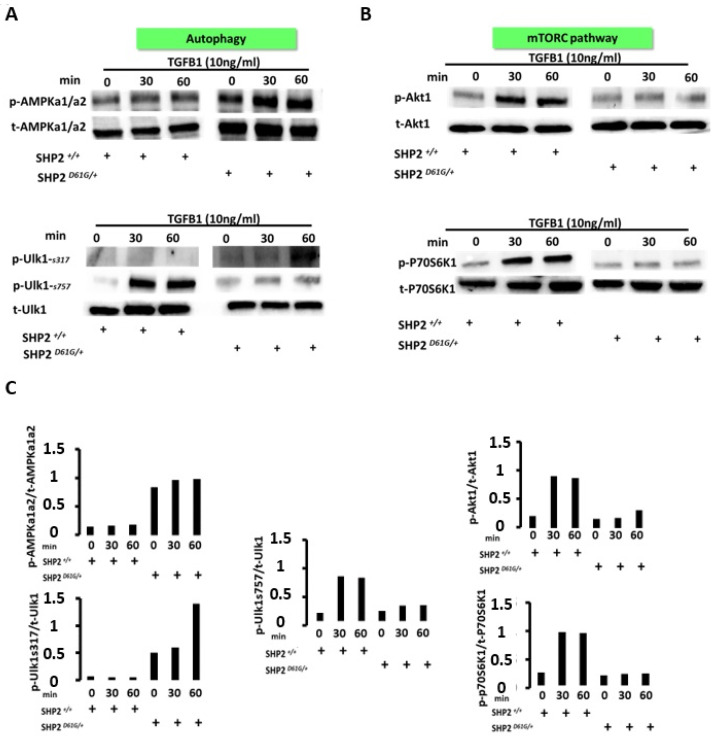
Immunoblot analysis showing that SHP2 positively regulates phosphorylation states of autophagy-related kinases and negatively regulates mTORC1 signaling pathways. (**A**) Autophagy signaling pathway: SHP2 constitutive activation augments TGFB1-induced phosphorylation of AMPKa1/a2 while baseline SHP2 expression leads to dephosphorylation of AMPK serine/threonine kinase complex. SHP2-induced AMPK activation led to enhanced phosphorylation of Ulk-1 at serine 317 while it dephosphorylated Ulk-1 at serine 757. (**B**) mTORC1 signaling pathway: SHP2 constitutive activation in DG1G+ mutants was associated with reduced TGFB1-induced phosphorylation of Akt-1 and p-P70S6K1, indicating inhibition of mTORC1 signal transduction pathway. Each lane represents an individual cell preparation. (**C**) Immunoblot densitometry analysis of p-AMPK-a1/a2 normalized to total AMPK-a1/a2, p-Ulk-1-ser317, p-Ulk1-ser757 normalized to total Ulk-1, p-Akt-1 normalized to total Akt-1 and p-P70S6K1 normalized to total P70S6K1. Data are presented as bar graphs. Each bar represents an individual lane. Details for this figure are presented in Supplemental Data S4.

SHP2 constitutive activation enhances AMPK activity leading to autophagy-associated Ulk1 activation and downregulation of the mTORC signaling pathway. To decipher the mechanisms through which SHP2 impairs mitochondrial metabolism of MLFs leading to autophagy, we investigated the effects of SHP2 constitutive activation on serine/threonine signal transduction pathways involved in the autophagy-signaling machinery. Intriguingly, SHP2 activation augmented TGFB1-induced phosphorylation of AMPKa1/a2 while baseline SHP2 expression led to dephosphorylation of AMPK serine/threonine kinase complex (Figure 4A-upper panel). Furthermore, SHP2-induced AMPK activation was associated with phosphorylation of Ulk-1 at serine 317, while it dephosphorylated Ulk-1 at serine 757. The SHP2-induced Ulk1-AMPK interaction led to the activation of Ulk-1, a serine/threonine kinase that affects autophagosome fusion and is critical for the initiation of autophagy (Figure 4A-lower panel) [25,26]. Since phosphorylation of ser 757 in Ulk-1 is an mTORC-dependent phosphorylation site and it was found reduced in SHP2^D61G/+^ MLFs we next investigated the effects of SHP2 overactivation on mTORC-signaling machinery. In line with our previous findings, SHP2 constitutive activation was associated with the downregulation of mTORC1-related serine/threonine kinase signal transduction pathways, as indicated by the reduced phosphorylation status of Akt1 and P70S6K1 kinases (Figure 4B-upper and lower panel, respectively, Supplemental Data S4). The latter evidence further ties with the notion that SHP2 activation acts as an upstream regulator of both AMPK and mTORC pathways serving as regulator of both cell mass and energy metabolism.

## 4. Discussion

Our study suggests a novel role for SHP2 as an anti-fibrotic mediator though mechanisms that involve induction of autophagy-initiating kinases and negative regulation of mTORC1-related signal transduction pathways, leading to impaired mitochondrial metabolism and attenuated fibrotic responses in primary lung fibroblasts. Given our previously published observations that SHP2 overexpression attenuates fibrotic responses both in vivo and in vitro in experimental models of pulmonary fibrosis [12], as well as the fact that: (1) SHP2 also localizes within mitochondrial space and (2) the cardinal role of mitochondrial homeostasis of lung structural cells in lung fibrosis [13], we focused on the role of SHP2 as a regulator of mitochondrial metabolism in primary MLFs derived from mice carrying a conditional knock-in mutation (D61G/+) that renders the SHP2 catalytic domain constitutively active. After validating our previously published data showing that SHP2^D61G/+^ constitutively active mutants display attenuated fibrotic responses to profibrotic stimuli, we performed in-depth mitochondrial morphological analysis using transmission electron microscopy (TEM). We observed that SHP2^D61G/+^ MLFs displayed abnormal, swollen mitochondria with electro-lucent cristae and an increased number of autophagosomes. Morphological abnormalities were associated with functional abnormalities as indicated by reduced mitochondrial membrane potential, coupling efficiency, and oxygen consumption rate, as well as increased ROS. To decipher the mechanisms underlying the SHP2-induced morphological and functional abnormalities in MLFs, we examined the effects of SHP2 constitutive activation in major signal transduction pathways involved in autophagy and cellular metabolism. Interestingly, SHP2 constitutive activation downregulated PINK1 expression while enhanced AMPK activity leading to autophagy-associated Ulk1 activation and downregulation of the mTORC signaling pathway.

SHP2 is a ubiquitously expressed phosphatase that was recently implicated by our study group as a negative regulator of fibroblast homeostasis, and when overexpressed, exerts anti-fibrotic properties in experimental models [12]. Interestingly, we and others have shown that SHP2 is also a target of nintedanib, an FDA-approved anti-fibrotic compound for the treatment of pulmonary fibrosis that acts as a kinase inhibitor but also as a phosphatase activator [12,27]. To this end, the interest in inhibiting the aberrant activation of kinase-controlled pro-fibrotic signal transduction pathways has been recently reinforced. On the other hand, the role of metabolic aberrations of structural and immune cells in regulating cellular phenotypes leading to profibrotic and/or pro-inflammatory milieu has recently emerged in the field of lung fibrosis with major therapeutic implications [24,28]. In particular, our study group has recently shown that AECIIs from IPF lungs are prone to apoptosis due to a thyroid hormone (TH) deficiency, and TH supplementation therapy attenuated experimental lung fibrosis and reversed morphological and functional mitochondrial abnormalities in AECIIs though mechanisms that involved induction of major transcription coactivators of mitochondrial biogenesis and effective mitophagy [13].

The evidence that SHP2 has been discovered to localize not only in the plasma membrane but also within the mitochondrial intermembrane space acting as a nutrient sensor, metabolic regulator, and integral component of oxidative phosphorylation and autophagic machinery is of particular interest [18,19,29,30]; yet, its role in regulating cellular phenotypes through autophagy and metabolic reprogramming in the context of lung fibrosis is largely unknown. To this end, we discovered that SHP2-mediated blunted fibrotic responses were associated with significant morphological and functional abnormalities of mitochondria derived from primary MLFs bearing a knock-in mutation (D61G), rendering the catalytic domain of SHP2 constitutively active. In particular, ultrastructural morphological analysis using the TEM of MLFs revealed that SHP2^D61G/+^ constitutively active mutants exhibited swollen mitochondria with disrupted, electro-lucent cristae, and aggregates of autophagosomes, findings that were further validated by immunoblot analyses and immunofluorescence, revealing increased expression of autophagy markers. Interestingly, mitochondrial functional analysis using extracellular flux technology demonstrated that SHP2 knock-in MLFs exhibited decreased mitochondrial membrane potential, ATP production, and coupling efficiency, implicating dysregulated SHP2 activity in mitochondrial homeostasis. Another pathological component linked to mitochondrial dysfunction in SHP2 active mutant MLFs was the significantly increased production of ROS, in line with previous data [19]. Generally increased ROS have been described in the context of enhanced mitochondrial metabolism as assessed by increased mitochondrial membrane potential and ATP production [31]. Nonetheless, decreased mitochondrial membrane potential levels combined with decreased energy levels (ATP production), coupling efficiency, and increased ROS may indicate ineffective and/or disrupted mitochondrial metabolism. The latter could also be supported by reduced PINK1 protein expression levels in SHP2 constitutively active MLFs, considering that PINK1 deficiency may lead to ineffective mitophagy, meaning clearance of damaged mitochondria, thus leading to accumulation of autophagosomes within the cytoplasm and further production of ROS. PINK1 deficiency-induced mitochondrial function derangements have been associated with enhanced AECIIs apoptosis [14] and reduced fibroblasts-to-myofibroblasts differentiation [32]. The above events may exert deleterious effects on fibroblast homeostasis and thus explain the observed proliferation arrest, reduced migratory, differentiative, and secretory capacity in response to pro-fibrotic stimuli such as TGFB1 and PDGF-BB.

The pathways by which SHP2 mediates its antifibrotic activity and regulates mitochondrial metabolism and mitophagy are of major interest. We provide significant evidence that SHP2 activation negatively regulates mitochondrial metabolism and induces mitophagy through regulation of kinase-controlled signal transduction pathways including AMPK and mTORC. In particular, we have demonstrated that SHP2 acts as an AMPK activator mediating inhibition of the mTORC pathway, leading to dephosphorylation of Ulk-1 in ser 757 and thus allowing interaction between AMPK-Ulk1, as previously shown [25]. AMPK then phosphorylates Ulk1 in ser317, leading to activation of Ulk1, an autophagy-initiating kinase. The activation of AMPK by SHP2 leading to initiation of the autophagic signaling machinery has been previously shown in 3T3 fibroblasts, where SHP2 inhibited the mTOR/S6K1 pathway through an AMPK-dependent manner, exerting a negative impact on cell size and energy status [33]. Similarly, herein ultrastructural TEM analysis revealed that SHP2^D61G/+^ constitutively active MLFs exhibited reduced cell size with diminished extracellular matrix production in response to TGFB1 treatment compared to wild-type ones. Our data support the notion that SHP2 dysregulated activity is responsible for impaired fibroblast mitochondrial bioenergetics and reduced energy production, leading to proliferative arrest as well as reduced migratory, differentiative, and secretory capacity. It is evident that SHP2 shifts metabolic programming towards low energy production (mimicking conditions of glucose starvation where AMPK is activated) and this may exert a negative impact on cellular homeostasis and bioenergetics. This evidence holds potential therapeutic promise in light of the beneficial effects of metformin as an anti-fibrotic mediator through AMPK activation [34]. Finally, it is also interesting that another mediator of cancer, SHP2, is a potential therapeutic target for pulmonary fibrosis [35,36,37]. Focusing on commonalities between cancer and pulmonary fibrosis might lead to novel anti-fibrotic compounds, similarly with the process of nintedanib repositioning [38].

Our study has a number of limitations that need to be treated with caution. Our report does not include in-vivo or ex-vivo data on the role of SHP2 in regulating metabolic perturbations of lung fibroblasts within the fibrotic microenvironment. In addition, further in-vitro read-out assays are needed to support the role of SHP2 as a regulator of fibroblast mitochondrial metabolism, including not only knock-in but also knock-out experiments. Furthermore, we do not present human data that could enhance the clinical applicability of our findings. Nevertheless, we have previously published rigid data that SHP2 is downregulated in IPF lungs and acts as an anti-fibrotic mediator in experimental models of lung fibrosis [12]. In terms of mechanisms and considering that SHP2 is a ubiquitously expressed phosphatase, it is unlikely that our data represent a cell-specific phenomenon while there are also several other SHP2-independent pathways that regulate AMPK activation and mTORC deactivation, as it has been previously shown [25,26,34]. A mechanistic linearity that associates attenuated fibrotic responses with a SHP2-dependent regulation of autophagy and mitochondrial homeostasis is also missing. Further studies will be required to further delineate the role of SHP2 as an anti-fibrotic mediator through the regulation of fibroblast homeostasis and mitophagy.

## 5. Conclusions

In conclusion, we provide for the first time in the context of lung fibrosis, strong in vitro evidence that SHP2 exerts its anti-fibrotic properties through negative regulation of mitochondrial metabolism and induction of mitophagy through mechanisms that involve interaction with metabolic kinase-controlled signal transduction pathways. Enhancing SHP2 activity within a profibrotic microenvironment may exert therapeutic effects by modulating metabolic perturbations, as it happens with AMPK activators.

## Data Availability

Data available upon request to the corresponding author.

## Supplementary Materials

Please add the following supporting information can be downloaded at: https://www.mdpi.com/article/10.3390/diagnostics13061166/s1, Supplemental Data S1–S4: Further details for the results presented in figures can be found in Supplemental Data S1–S4.
