# Supplementary material for: SH2 Domain-Containing Phosphatase-SHP2 Attenuates Fibrotic Responses through Negative Regulation of Mitochondrial Metabolism in Lung Fibroblasts
Source: Diagnostics (Basel). 2023 Mar 18;13(6):1166. doi: 10.3390/diagnostics13061166 (PMC10047203; doi:10.3390/diagnostics13061166)
Supplement: Supplementary file 1 [file diagnostics-13-01166-s001.zip › diagnostics-2280910-supplementary.pdf]

Supplemental data

SH2 domain-containing phosphatase-SHP2 attenuates fibrotic responses through negative regulation of mitochondrial metabolism in lung fibroblasts

Supplemental Data S1 (Figure 1). SHP2 D61G/+ constitutively active mutant negatively regulates fibroblast migration, myofibroblast differentiation and proliferation. A) Primary mouse lung fibroblasts (MLF) from SHP2D61G/+ (constitutively active mutants) and SHP2+/+ mice were seeded in 24-well plates at 2 × 105 cells/mL and incubated for 24h. After cells reached 100% confluence, wounds were generated using a 1 mL micropipette tip. Media was removed, cells washed with 500 µL PBS, and 500 µL of complete culture media added into each well. Images were acquired immediately following media replacement (T = 0), and every 6 h for 24 h via multi-mode plate reader at 10×. After exporting images, wound areas were measured using ImageJ. B) Extents of closure at T6, T12, T18, and T24 were calculated by subtracting area at T0; percentage closure was determined by normalizing difference to area at T0. SHP2 D61G/+ constitutively active mutant lung fibroblasts exhibited significantly reduced cell migration at all time points (T0-T24, 2-fold) compared to wild type ones (SHP2+/+). One way ANOVA, *p<0.05. Data represent mean + SD of 6 samples (biological replicates). C) Double immunofluorescence analysis in representative primary MLF samples showing decrease in TGFB1-induced a-SMA (red) and stress fibers as indicated by phalloidin green (merged-yellow) in SHP2 D61G/+ constitutively active mutant lung fibroblasts compared to wild type ones (SHP2+/+). D) Primary mouse lung fibroblasts (MLF) from SHP2D61G/+ (constitutively active mutants) and SHP2+/+ mice were stimulated with PDGF-BB (25ng/ml) or mock (PBS) for 6 hrs. PDGF-BB stimulation induced a significant increase (1.4-fold) in proliferation rates of SHP2+/+ MLFs compared to the SHP2D61G/+ constitutively active mutants. Each bar represents mean expression of 6 samples (biological replicates). Data (absorbance) represent mean + SD. One way ANOVA, *p<0.05.

Supplemental Data S2 (Figure 2). SHP2 D61G/+ constitutively active mutant mouse lung fibroblasts (MLFs) display increased expression of autophagy markers, swollen mitochondria with disrupted cristae and increased number of au-tophagosomes. A) Transmission Electron microscopy (TEM) of SHP2 D61G/+ constitutively active mutant MLFs compared to wild type ones (SHP2+/+) before and after treatment with TGFB1 (10ng/ml for 6 hrs) showed that SHP2 constitutive activation was associated with increased number of autophagosomes and swollen mitochondria with disrupted, electro-lucent cristae (white ar-rows-insets). Note the shrinked cytoplasm and the absence of extracellular matrix production in SHP2 D61G/+ constitutively active mutants which is evident in wild type MLFs ((SHP2+/+) (white arrow heads), following treatment with TGFB1. Representative images of 6 samples (biological replicates). B) Immunoblot analyses of autophagy markers (LC3BI/II) and autophagy receptor (p62) showed a LC3Bhighp62low immunoblot pattern in SHP2 D61G/+ constitutively active mutant MLFs compared to wild type ones (SHP2+/+), indicating increased autophagy. C) Double immunofluo-rescence analysis in MLFs validated immunoblot results showing co-localization (yellow-merged) of autophagy marker LC3B (green) with mitotracker (red stain of active mitochondria) in SHP2 D61G/+ constitutively active mutant MLFs. D) Surprisingly immunoblot analysis of PINK1 (mediator of effective autophagy) showed decreased expression in SHP2 D61G/+ constitutively active mutant MLFs compared to wild type ones (SHP2+/+), potentially indicating ineffective autophagy. E) Im-munoblot densitometry analysis of LC3BI/II, p62 and PINK1 normalized to β-actin. Data are pre-sented as bar graphs. Each bar represents an individual lane.

Supplemental Data S3 (Figure 3). SHP2 D61G/+ constitutively active mutant mouse lung fibroblasts exhibit impaired mitochondrial function with increased ROS production. A) Mitochondrial function SHP2 $^{D61G/+}$ constitutively active mutant mouse lung fibroblasts (MLFs) in cultured SHP2 $^{D61G/+}$ constitutively active mutant MLFs compared to wild type ones (SHP2$^{+/+}$). (B) Maximum respiration, (pmol/min) was measured under basal conditions followed by addition of oligomycin, FCCP, rotenone and antimycin as indicated, $*P < 0.05$. C) Coupling efficiency (ATP production/proton leak), a measure of effective mitochondrial metabolism in SHP2 $^{D61G/+}$ constitutively active mutant MLFs compared to wild type ones (SHP2$^{+/+}$). D) Reactive oxygen species (ROS) were measured using the probe 5-(and-6)-chloromethyl-2′,7′-dichlorodihydrofluorescein diacetate acetyl ester (CM-H2DCFDA). Compared to wild type ones, D61G cells show a 1.6-fold increased fluorescence indicating significantly increased ROS levels Note that SHP2 $^{D61G/+}$ constitutively active MLFs were characterized by reduced MMP levels (1.3-fold), maximum respiration (oxygen consumption rate) (1.86 fold) and coupling efficiency (1.9-fold) indicating ineffective mitochondrial metabolism potentially resulting to increased ROS (1.6-fold) compared to wild type ones. Data are presented as bar graphs with horizontal bars representing mean mitochondrial membrane potential (MMP) levels (green/red fluorescence ratio) $\pm$ SEM, of 6 samples (biological replicates). Independent samples Student's t-test, $*P < 0,05$.

Supplemental Data S4 (Figure 4). SHP2 positively regulates phosphorylation states of autophagy-related and negatively regulates mTORC1 signaling pathways. A) Autophagy signaling pathway: Upper panel: Immunoblot analyses for p-AMPK-a1/a2 and total AMPK-a1/a2 at different time-points (0, 30, 60 min) following TGFB1 stimulation (10ng/ml). Note that SHP2 constitutive activation augments TGFB1-induced phosphorylation of AMPKa1/a2 while baseline SHP2 expression leads to dephosphorylation of AMPK serine/threonine kinase complex. Lower panel: Immunoblot analyses for p-Ulk-1-ser317, p-Ulk1-ser757 and total Ulk-1 at different time-points (0, 30, 60 min) following TGFB1 stimulation (10ng/ml). Each lane represents an individual cell preparation. Note that SHP2-induced AMPK activation led to enhanced phosphorylation of Ulk-1 at serine 317 while dephosphorylated Ulk-1 at serine 757. B) mTORC1 signaling pathway: Upper panel: Immunoblot analyses for p-Akt1 and total Akt1 at different time-points (0, 30, 60 min) following TGFB1 stimulation (10ng/ml). Lower panel: Immunoblot analyses for p-P70S6K1 and total P70S6K1 at different time-points (0, 30, 60 min) following TGFB1 stimulation (10ng/ml). SHP2 constitutive activation in DG1G+ mutants was as-sociated with reduced TGFB1-induced phosphorylation of Akt-1 and p-P70S6K1, indicating inhi-bition of mTORC1 signal transduction pathway. Each lane represents an individual cell prepara-tion. C) Immunoblot densitometry analysis of p-AMPK-a1/a2 normalized to total AMPK-a1/a2, p-Ulk-1-ser317, p-Ulk1-ser757 normalized to total Ulk-1, p-Akt-1 normalized to total Akt-1 and p-P70S6K1 normalized to total P70S6K1. Data are presented as bar graphs. Each bar represents an individual lane.
